# Untargeted metabolomics in gastric and colorectal cancer patients – preliminary results

**DOI:** 10.3389/fcimb.2024.1394038

**Published:** 2024-05-07

**Authors:** Karolina Kaźmierczak-Siedlecka, Damian Muszyński, Daniel Styburski, Jakub Makarewicz, Bartosz Kamil Sobocki, Paweł Ulasiński, Karol Połom, Ewa Stachowska, Karolina Skonieczna-Żydecka, Leszek Kalinowski

**Affiliations:** ^1^ Department of Medical Laboratory Diagnostics – Fahrenheit Biobank BBMRI.pl, Medical University of Gdansk, Gdansk, Poland; ^2^ Scientific Circle of Studies Regarding Personalized Medicine Associated with Department of Medical Laboratory Diagnostics, Medical University of Gdansk, Gdansk, Poland; ^3^ Sanprobi Sp. z o. o., Szczecin, Poland; ^4^ Department of Oncology and Radiotherapy, Medical University of Gdansk, Gdansk, Poland; ^5^ Unit of Surgery with Unit of Surgery with Unit of Oncological Surgery, Specialist Hospital in Koscierzyna, Koscierzyna, Poland; ^6^ Academy of Medical and Social Applied Sciences, Elbląg, Poland; ^7^ Department of Surgical Oncology, Medical University of Gdansk, Gdansk, Poland; ^8^ Department of Gastrointestinal Surgical Oncology, Greater Poland Cancer Centre, Poznan, Poland; ^9^ Department of Human Nutrition and Metabolomics, Pomeranian Medical University in Szczecin, Szczecin, Poland; ^10^ Department of Biochemical Research, Pomeranian Medical University in Szczecin, Szczecin, Poland; ^11^ BioTechMed Centre/Department of Mechanics of Materials and Structures, Gdansk University of Technology, Gdansk, Poland

**Keywords:** colorectal cancer, gastric cancer, gut microbiome, microbiota-derived metabolites, untargeted metabolomics

## Abstract

**Introduction:**

Recent years, microbiota-associated aspects have been analysed in multiple disorders regarding cancers. Existing evidence pints that gut microorganisms might take part in tumour origin and therapy efficacy. Nevertheless, to date, data on faecal metabolomics in cancer patients is still strongly limited. Therefore, we aimed to analyse gut untargeted metabolome in gastrointestinal cancer patients (i.e., gastric and colorectal cancer).

**Patients and methods:**

There were 12 patients with either gastric (n=4) or colorectal cancer (n=8) enrolled and 8 analysed (n=4 each). Stool samples were collected prior to anti-cancer treatments. Untargeted metabolomics analyses were conducted by means of mass spectrometry.

**Results:**

A plethora of metabolites in cancer patients we analysed were noted, with higher homogenity in case of gastric cancer patients. We found that the level of Deoxyguanosine,m/z 266.091,[M-H]-, Uridine,m/z 245.075,[M+H]+, Deoxyguanosine,m/z 268.104,[M]+, 3-Indoleacetic acid,m/z 176.07,[M+H]+, Indoxyl,m/z 132.031,[M-H]-, L-Phenylalanine,m/z 164.073,[M-H]-, L-Methionine,m/z 150.058,[M+NH4]+, was significantly higher in colorectal cancer patients and Ethyl hydrogen malonate,m/z 133.031,[M+H]+ in gastric cancer.

**Conclusion:**

The overall insights into untargeted metabolomics showed that most often higher levels of analysed metabolites were detected in colorectal cancer patients compared to gastric cancer patients. The link between gut metabolome and both local and distal metastasis might exist, however it requires confirmation in further multi-centre studies regarding larger sample size.

## 1 Introduction

Microbiome and metabolome-related aspects have become objects of interest in oncology (Kaźmierczak-Siedlecka et al., 2023). The reasons are as follows: [1] Currently, it is known that some microbes are involved in development of tumour by creating dysbiotic environment and activating biochemical pathways (Rajagopala et al., 2017). There are therapeutic methods (such as prebiotics, probiotics, synbiotics, postbiotics, next-generation probiotics) which modify the composition of gut microbiome and the activity of microorganisms through for instance affecting production of metabolites and consequently leading to eubiosis restoration. However, it is still under investigation, and it requires further analysis to strengthen the possibility of usage. [2] According to some data, there is a bidirectional link between gut microbiome and drugs (also anti-cancer agents). These interactions are described as pharmacomicrobiomics (Ting et al., 2022). Basis on this bidirectional communications may provide personalized and more effective anti-cancer management. [3] Microbiome profile and metabolomic signature may be considered as biomarkers (Wong and Yu, 2023), which can select subjects with higher risk of tumour development or to detect cancer in early stages. Therefore, it seems that there can be found many benefits from routinely analysis of gut microbiome in cancer patients and include it to screening program.

In contrast to targeted metabolomics, untargeted metabolomics is characterized by wide range of discovery, mainly hypothesis generating, comprehensive analysis, qualitative identifications and relative quantitation of small molecules in sample (Schrimpe-Rutledge et al., 2016). In the level of metabolomics, small molecules are characterized from many types of samples, such as stool, urine, serum, cell extracts, and others. Considering metabolomics it should be emphasized that there are different methods of both separation and detection. Notably, it seems that metabolomics analysis based on mass spectrometry is one of the most significant technology allowing to detect and identify small molecules which are produced by gut microbiota (Bauermeister et al., 2022).

As it was previously mentioned, the imbalance of gut microbiota composition and changes of microbiota-derived metabolites are observed in gastrointestinal cancer patients (Kaźmierczak-Siedlecka et al., 2023; Ohigashi et al., 2013; Tong et al., 2021; Yang et al., 2022; Dai et al., 2021). Recently, in Kaźmierczak-Siedlecka et al. study it was shown that microbiota-derived metabolites based on the proportion between acetate, proprionate, and butyrate is changed in colorectal cancer patients in preoperative period (Kaźmierczak-Siedlecka et al., 2023). Untargeted metabolomics seems to be extremely significant in oncology due to the fact that it allows to collect data without pre-existing knowledge (Schrimpe-Rutledge et al., 2016). It is noteworthy that anti-cancer treatment (such as surgery, chemotherapy, radiotherapy) affects gut microbiome and metabolome-related aspects and vice-versa. Therefore, the aim of this study was to analyse untargeted metabolomics in patients with gastrointestinal cancers (i.e. gastric cancer and colorectal cancer) prior to the introduction of anti-cancer treatment. It allows to obtain more precise data without the potential influence of above mentioned treatment. Moreover, the comparison of untargeted metabolomics in case of gastric and colorectal cancer has been investigated.

## 2 Patients and methods

Participants (n=12) were recruited in Department of Surgical Oncology (Medical University of Gdansk) and Unit of Surgery with Unit of Surgery with Unit of Oncological Surgery, Specialist Hospital in Koscierzyna, Poland. Inclusion criteria were age ≥18 yr., patients with diagnosed gastric/colorectal cancer prior to the introduction of anti-cancer treatment, written consent to take part in this study. Exclusion criteria included age <18 yr., patients with gastric/colorectal cancer who were under anti-cancer treatment. The stool samples (at least 4 g) were collected after confirming of diagnosis and before introduction of anti-cancer treatment. The stool samples were taken by own patients, placed in sterile tube, and then provided to researchers as soon as possible. Next, they were stored in -80°C in the Fahrenheit Biobank BBMRI.pl, Medical University of Gdansk, until conduction of untargeted metabolomics analysis according to the well-established protocol at Sanprobi Sp. z o. o. The study protocol has been approved by the Independent Bioethics Committee for Scientific Research at the Medical University of Gdansk (identifiers: NKBBN/129/2021, NKBBN/428/2022, KB/428-314/2023).

### 2.1 Preparation of material for analysis

Briefly, 500 µl of a mixture of methanol, water and acetonitrile in the proportions of 50:25:25 v/v/v with the addition of deuterated internal standards was added to 60 mg of feces. Then, the samples were shaken at 2000 rpm at 4°C for 30 min. to dissolve the metabolites in the solution and precipitate the proteins. In the next step, the samples were centrifuged for 4 minutes at a speed of 4000 rpm and at a temperature of 4°C. After the samples were centrifuged, the supernatant was decanted to the chromatography tubes through a 0.22 μm syringe filter. The samples were subsequently analysed on the same day by a liquid chromatography–mass spectrometry. QC samples were prepared by mixing test samples in equal proportions and prepared in the same way as the test samples.

### 2.2 Liquid chromatography-mass spectrometry analysis

The analysis was carried out on an ExionLC liquid chromatograph equipped with a binary pump, autosampler, and column thermostat coupled with a Triple TOF 6600+ mass spectrometer (Sciex, Framingham, MA, USA). The separation was carried out on a Phenomenex Luna^®^ Omega 1.6μm polar C18 150 x 2.1mm column for 45 min in gradient separation. The mobile phases were: Phase A – Water with 10mM ammonium acetate, Phase B - acetonitrile with 0.1% formic acid. The column injection was 2μl and the column temperature was 20°C. The phase flow was 0.2 ml/min. Spectral analysis was performed in the positive ion mode with a capillary voltage of 5500 V, Curtain gas (CUR) was 25 psi, Ion source gas 1 (GS1) 45 psi, Ion source gas 2 (GS2) 60 psi and the ion source temperature was 400°C and the mode negative ions at a capillary voltage of 4500 V, Curtain gas (CUR) was 25 psi, Ion source gas 1 (GS1) 45 psi, Ion source gas 2 (GS2) 60 psi and the ion source temperature was 350°C. Spectrometer collected spectral data in SWATH mode.

### 2.3 Analysis of the results and statistical analysis

The obtained spectral spectra were analysed and matched to reference spectra contained in the SCIEX All-In-One HR-MS/MS, NIST and own databases using SCIEX OS software. In the next step, based on the results obtained and the identification of metabolites present in the tested samples, a file was created in Microsoft Excel 2019 PL (Poland) for statistical analysis and data visualization on the Metaboanalyst platform (https://www.metaboanalyst.ca/). The t-test and fold change >2 were used to determine differences between the study groups. The statistical analysis was conducted using above mentioned Microsoft Excel 2019 PL (Poland) and STATISTICA version 13.0.

## 3 Results

This study included 12 patients (n=8 – colorectal cancer, n=4 – gastric cancer). The basic characteristics of these participants is as follows: the median age – 61.78 ± 11.50 years, the median Body Mass Index (BMI) – 29 ± 1.41 kg/m^2^, the most commonly co-existing disease – hypertension. Among these patients, 4 were excluded due to incomplete data regarding tumour characteristics. Therefore, the analysis is based on 2 groups: first including gastric cancer patients (n=4) and second regarding colorectal cancer patients (n=4) (**Table 1**).

**Table 1:** Characteristics of patients according to the tumour types.

Sample_1G	Stomach cancer T3N0M0
Sample_N2G	Stomach cancer NET, G1
Sample_3G	Cancer of the prepyloric part of the stomach T3N1M0
Sample_4G	Stomach cancer T2N1
Sample_5G	Cancer of the sigmoid-rectal flexure pT3N2a
Sample_9G	Sigmoid colon cancer adenocarcinoma G2 cT4NxM1b
Sample_13AG	Rectal cancer – adenocarcinoma G2 pT2N0M0
Sample_20AG	Ascending colon cancer pT2N0M0

The analysis of stool samples revealed the occurrence of wide range of metabolites in gastric and colorectal cancer patients (**Table 2**).

**Table 2:** Metabolites identified in analysed stool samples of gastric and colorectal cancer patients.

Compound	Precursor Mass	Adduct	Retention time
Enterolactone	297.115	[M-H]-	23.5
(2-Oxo-2,3-dihydro-1H-indol-3-yl)acetic acid	192.064	[M+H]+	16.8
(2-Oxo-2,3-dihydro-1H-indol-3-yl)acetic acid	190.051	[M-H]-	16.8
Gamma-Undecalactone	185.152	[M+H]+	23.3
1,1-Dimethylbiguanide	130.109	[M+H]+	5.2
1,3,7-Trimethyluric acid	211.082	[M+H]+	16.8
1,3,7-Trimethyluric Acid	209.069	[M-H]-	16.8
1,7-Dimethyluric Acid	197.066	[M+H]+	16.0
1,7-Dimethyluric Acid	195.053	[M-H]-	16.0
1,9-Nonanedicarboxylic acid	215.13	[M-H]-	22.5
12-Hydroxystearic Acid	301.273	[M+H]+	30.4
17.alpha.-Ethyl-5.beta.-estrane-3.alpha.,17.beta.-diol	289.252	[M+H]+	29.1
17a-Ethynylestradiol	295.167	[M-H]-	16.3
1-Aminocyclohexanecarboxylic acid	144.101	[M+H]+	3.4
1-Methyl-1H-purine-2,6(3H,7H)-dione	167.055	[M+H]+	15.5
1-Methyl-1H-purine-2,6(3H,7H)-dione	165.042	[M-H]-	15.6
1-Methyl-4-imidazoleacetic Acid	141.065	[M+H]+	2.4
1-Methyluric Acid	183.05	[M+H]+	9.2
2,2'-Methylene-bis(6-tert-butyl-4 methylphenol)	339.234	[M-H]-	32.3
2,8-Quinolinediol	160.041	[M-H]-	18.9
2,8-Quinolinediol	162.054	[M+H]+	18.9
2-Hydroxy Stearic Acid	299.261	[M-H]-	30.4
2-Hydroxy-3-methoxybenzaldehyde	151.027	[M-H]-	10.1
2-Hydroxyhexadecanoic Acid	271.228	[M-H]-	31.6
2-Methoxymethcathinone	194.117	[M+H]+	20.4
2-Methyl-3-ketovaleric acid	129.056	[M-H]-	11.8
2-Oxindole	134.06	[M+H]+	19.8
2-Phenylbutyric acid	165.09	[M+H]+	9.0
2-Phenylglycine	150.043	[M-H]-	14.9
2-Piperidinone	100.076	[M+H]+	14.9
3b-Hydroxy-5-cholenoic acid	373.276	[M-H]-	31.8
3b-Hydroxy-5-cholenoic acid	419.282	[M+FA-H]-	31.8
3-Hydroxydodecanoic acid	215.166	[M-H]-	25.3
3-Indoleacetic acid	176.07	[M+H]+	20.2
3-Nitrotyrosine	227.081	[M+H]+	18.5
3β-Ursodeoxycholic Acid	391.287	[M-H]-	25.3
4-Methyl-5-thiazoleethanol	144.046	[M+H]+	17.4
5-Aminovaleric acid	116.073	[M-H]-	2.4
7(S),17(S)-Dihydroxy-8(E),10(Z),13(Z),15(E),19(Z)-docosapentaenoic acid	345.237	[M+H]+	30.4
7-Methylguanine	166.072	[M+H]+	15.0
9E,11E-Octadecadienoic acid	281.247	[M+H]+	31.0
Adenine	136.061	[M+H]+	15.2
Aminocaproic acid	130.088	[M-H]-	4.5
Arachidonic Acid	303.234	[M-H]-	32.4
Argininosuccinic acid	291.145	[M+H]+	18.5
Azelaic acid	187.099	[M-H]-	9.2
Benzoic acid	121.03	[M-H]-	19.7
Beta-N-Acetylglucosamine	222.097	[M+H]+	2.4
Biocytin	371.191	[M-H]-	22.0
Biotin	245.095	[M+H]+	17.3
Butyric acid	87.046	[M-H]-	3.2
Cholesterol sulfate	465.306	[M-H]-	31.8
cis-4,10,13,16-Docosatetraenoic acid	331.266	[M-H]-	33.7
cis-4,7,10,13,16,19-Docosahexaenoic acid	327.234	[M-H]-	32.1
cis-5,8,11-Eicosatrienoic acid	305.25	[M-H]-	33.2
Citrulline	176.102	[M+H]+	2.2
Citrulline	174.089	[M-H]-	2.2
Curcumin	369.133	[M+H]+	26.3
Delta-Hexanolactone	115.074	[M+H]+	4.7
Deoxyguanosine	268.104	[M]+	15.2
Deoxyguanosine	266.091	[M-H]-	15.2
Deoxyinosine	253.092	[M+H]+	15.0
Deoxyinosine	251.08	[M-H]-	15.0
D-Glutamine	145.063	[M-H]-	2.0
Dimethylglycine	102.057	[M-H]-	1.8
D-Mannose	179.057	[M-H]-	2.1
Dodecanedioic acid	229.146	[M-H]-	23.3
Dodecanedioic acid	251.128	[M+Na-2H]-	23.3
Dodecanedioic acid	248.185	[M+NH4]+	23.3
Dodecanoic acid	199.171	[M-H]-	29.9
D-Xylitol	151.062	[M-H]-	2.1
Ethyl hydrogen malonate	133.031	[M+H]+	3.4
Geranyl caprylate	303.231	[M+H]+	28.8
Glutaric acid	131.035	[M-H]-	1.8
Glycodeoxycholic Acid	448.308	[M-H]-	24.0
Glycolithocholic Acid	432.313	[M-H]-	27.0
Guanidinosuccinic acid	174.041	[M-H]-	1.8
Guanosine	284.099	[M+H]+	14.9
Hippuric acid	178.056	[M-H]-	15.3
Hydrocinnamic acid	149.062	[M-H]-	21.7
Hyocholic Acid	409.314	[M+H]+	26.2
Hyodeoxycholic acid	391.287	[M-H]-	27.1
Hyodeoxycholic acid	410.326	[M+NH4]+	25.8
Ile-Ile	245.185	[M+H]+	15.3
Indole-6-carboxaldehyde	146.059	[M+H]+	21.3
Indoxyl	132.031	[M-H]-	1.8
Inosine	269.087	[M+H]+	14.8
Inosine	267.075	[M-H]-	14.8
Isoleukotoxin Diol	313.24	[M-H]-	26.2
Kaempferol	285.056	[M-H]-	14.5
L-Alanine	88.041	[M-H]-	2.0
L-Arginine	173.105	[M-H]-	2.2
L-Glutamic acid	148.06	[M+H]+	1.8
L-Glutamic acid	146.047	[M-H]-	1.8
Linoleic acid	279.234	[M-H]-	30.1
L-Isoleucine	132.101	[M+H]+	3.5
Lithocholic acid	375.292	[M-H]-	30.7
L-Leucine	132.101	[M+H]+	4.6
L-Leucine	132.101	[M+H]+	4.6
L-Lysine	145.099	[M-H]-	1.9
L-Methionine	150.058	[M+H]+	3.4
L-Phenylalanine	166.086	[M+H]+	12.1
L-Phenylalanine	164.073	[M-H]-	11.9
L-Proline	116.07	[M+H]+	2.3
L-Proline	114.057	[M-H]-	2.3
L-Tryptophan	203.083	[M-H]-	16.1
L-Tyrosine	182.08	[M+H]+	7.1
L-Tyrosine	180.067	[M-H]-	6.5
L-Valine	118.086	[M+H]+	2.3
Mandelic acid	151.027	[M-H]-	9.4
Methylcysteine	134.048	[M-H]-	15.1
Myristic acid	227.203	[M-H]-	32.0
N-Acetylglutamic acid	190.07	[M+H]+	1.8
N-Acetylglutamic acid	188.057	[M-H]-	1.8
N-Acetyl-L-phenylalanine	208.096	[M+H]+	16.8
N-Alpha-acetyllysine	187.109	[M-H]-	2.4
Nicotinic acid	124.038	[M+H]+	4.4
Nutriacholic Acid	391.284	[M+H]+	25.5
Nutriacholic Acid	389.271	[M-H]-	25.5
Nutriacholic Acid	413.266	[M+Na]+	25.5
Nutriacholic Acid	408.311	[M+NH4]+	25.5
Nα-Acetyl-L-lysine	189.122	[M+H]+	2.4
Oleic acid	281.25	[M-H]-	34.2
Oleic acid	327.255	[M+FA-H]-	34.2
Ornithine	131.083	[M-H]-	2.2
Palmitoylethanolamide	300.289	[M+H]+	30.4
Pantothenic acid	218.104	[M-H]-	7.5
Phenylacetic acid	135.046	[M-H]-	14.4
Phosphocreatine	212.054	[M+H]+	1.7
Pipecolic acid	130.085	[M+H]+	3.2
Piperine	286.143	[M+H]+	26.2
Pregnenolone	395.172	[M-H]-	18.3
Propane-1,2,3-tricarboxylic acid	175.026	[M-H]-	1.6
Propane-1,2,3-tricarboxylic acid	157.015	[M-H2O-H]-	1.6
Propane-1,2,3-tricarboxylic acid	177.038	[M+H]+	1.7
Pseudouridine	243.063	[M-H]-	4.7
Pyrrolidonecarboxylic acid	130.049	[M+H]+	1.8
Quinolin-2-ol	144.046	[M-H]-	21.3
Sebacic acid	201.114	[M-H]-	20.9
Sodium glycochenodeoxycholate	450.32	[M+H]+	24.1
Sphinganine	302.305	[M+H]+	27.6
Suberic acid	173.083	[M-H]-	5.7
Tetradecanedioic acid	257.177	[M-H]-	25.1
Tetraethylene glycol	195.122	[M+H]+	15.5
Theobromine	181.071	[M+H]+	16.1
Thymidine	243.096	[M+H]+	15.5
Thymidine	241.084	[M-H]-	15.5
Thymine	127.049	[M+H]+	11.6
Thymine	125.036	[M-H]-	11.4
Aconitic acid	172.995	[M-H]-	1.9
Tyramine	138.091	[M+H]+	12.8
Uracil	113.034	[M+H]+	4.8
Uracil	111.02	[M-H]-	4.8
Uridine	245.075	[M+H]+	10.3
Urocanic acid	139.05	[M+H]+	3.2
Ursodeoxycholic acid	410.327	[M+H]+	27.2
Linoleic acid	325.239	[M+FA-H]-	32.8
2-Hydroxyadenosine	282.086	[M-H]-	14.9
Hederagenin	471.349	[M-H]-	27.8
Kaurenoic acid	301.203	[M-H]-	23.0
6-Hydroxypurine	135.032	[M-H]-	9.5
Betulinic acid	455.354	[M-H]-	33.1
Asiatic acid	487.344	[M-H]-	24.9
Adenosine	268.104	[M+H]+	15.8
Theophylline	181.071	[M+H]+	16.8
Peiminine	430.331	[M+H]+	21.2
Peimine	432.347	[M+H]+	22.5
Ginkgolic Acid	345.244	[M-H]-	34.2
Indirubin	261.078	[M-H]-	21.2

**Figure 1:**
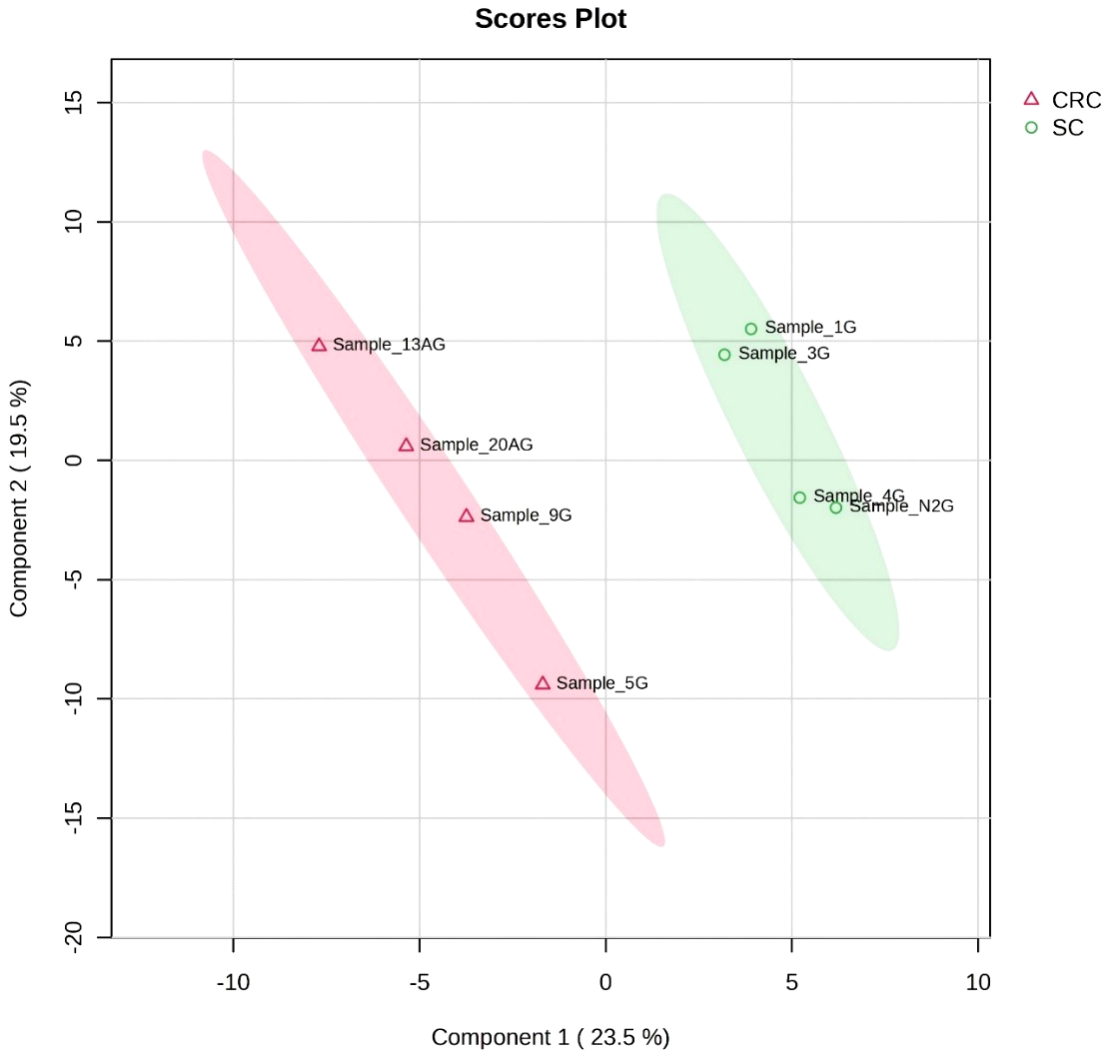
Figure PLS_DA – the comparing analysed groups – gastric cancer [SC] and colorectal cancer [CRC]. SC, stomach cancer; CRC, colorectal cancer.

The metabolic profile of analysed stool samples varies, especially in case of colorectal cancer patients (**Figure 1**). These differences can be caused by variability of either types of tumours or tumours anatomical localisation. There is higher grouping in case of gastric cancer, which confirms more homogeneous metabolic profile comparing to the analysed group of colorectal cancer. Moreover, in **Figure 1** there are subgroups (in gastric cancer) created by Sample_1G and Sample_3G, Sample_4G and Sample_N2G, which show similar characteristics in these subgroups.

**Figure 2:**
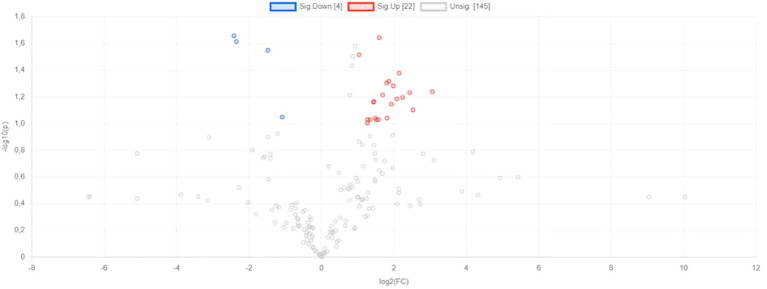
Volcano plot presenting metabolites varied groups of gastric cancer [SC] and colorectal cancer [CRC]. Significance parameters: p<0.1; FC > 2.

**Figure 3:**
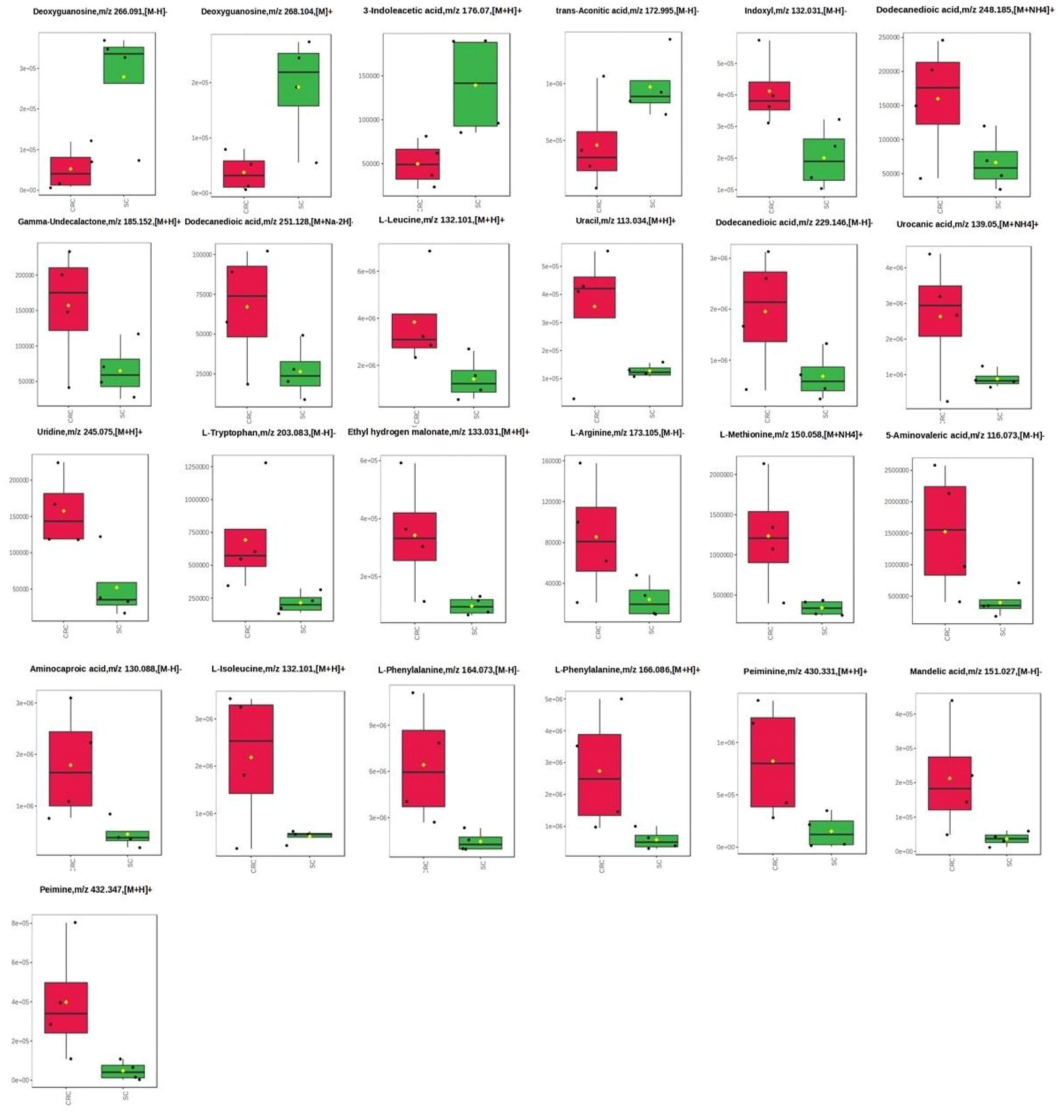
The levels of metabolites among SC and CRC.

The occurrence of metabolites, which varied in both analysed groups, is presented in **Figure 2**. The metabolites, which significantly varied colorectal cancer and gastric cancer are placed in **Figure 2** with blue and red colours and next they are precisely analysed and presented in **Figure 3**.

The comparison of the levels of particular metabolites detected in colorectal cancer patients and gastric cancer has been presented in **Figure 3**. Considering 25 metabolites (**Figure 3**), it is observed that higher level of them are mostly noted in colorectal cancer patients compared to the gastric cancer (21 metabolites *vs*. 4 metabolites, respectively). For instance, the levels of L- Leucine, L- tryptophan, L-Phenylalanine are higher in colorectal cancer than in gastric cancer. Moreover, considering extremely precise statistical condition, the statistically significant difference (p<0.05 and FC – 2) between analysed groups were found in case of Deoxyguanosine,m/z 266.091,[M-H]-, Uridine,m/z 245.075,[M+H]+, Deoxyguanosine,m/z 268.104,[M]+, 3-Indoleacetic acid,m/z 176.07,[M+H]+, Indoxyl,m/z 132.031,[M-H]-, L-Phenylalanine,m/z 164.073,[M-H]-, L-Methionine,m/z 150.058,[M+NH4]+, Ethyl hydrogen malonate,m/z 133.031,[M+H]+.

## 4 Discussion

Molecular diagnosis of cancer based on metabolomics can be promising in near future (Cheung et al., 2019). Metabolomic data may be used as biomarkers allowing to detect several cancers, such as oesophageal, gastric, pancreatic, bladder, lung, thyroid, and others (Wang et al., 2022; Yang et al., 2022). Different metabolites/metabolic pathways/metabolism may provide a signature which is specific for diseases/conditions. For instance, in a study by Yang et al., it was noted that glycophospholipid metabolism is related to both tumorigenesis and progression of oesophageal squamous cell carcinoma (ESCC) and that may be therapeutic target in ESCC progression (Yang et al., 2022). Hang et al. reported that untargeted plasma metabolomics can serve as a potential risk prediction of hepatocellular carcinoma (Hang et al., 2022). The aspects of untargeted metabolomics can be also useful in case of other digestive cancers. Plasma metabolomic signatures in precancerous gastric lesions progressing to cancer were identified in a study by Huang et al. (2021). Notably, six plasma metabolites were related to the both overall risk of gastric cancer and early gastric cancer whereas three of these metabolites, such as α-linolenic acid, linoleic acid, palmitic acid were associated with the prediction of risk of gastric lesion progression and early gastric cancer. In another study untargeted metabolome was also analysed in case of gastric cancer (Yu et al., 2021). Serum samples were taken from patients with chronic gastritis/gastric cancer. It was shown that lipid metabolism may affect the development of chronic gastritis to gastric cancer; moreover, hexadecasphinganine, linoleamide, and N-Hydroxy arachidonoyl amine were assessed as diagnostic markers for both chronic gastritis and gastric cancer (Yu et al., 2021). In the current study, we also investigated gut metabolome in cancer patients, but from stool samples. The overall insights showed that higher level of analysed metabolites was mostly noted in colorectal cancer patients compared to gastric cancer patients. For instance, in case of indole-3-acetic acid and tryptophan, the levels are higher in colorectal cancer than in gastric cancer. Indole-3-acetic acid is a tryptophan metabolite produced by gut microbiota according to the following pathway in intestinal epithelial cells: (1) ingested dietary protein, (2) tryptophan, (3) intestinal microbiota, (4) indole-3-acetic acid (Seo and Wargo, 2023; Tomii et al., 2023). This result can be associated with different overall characteristics of gut microbiota in particular types of cancer, i.e. gastric and colorectal cancer. In the current study, it was observed that the level of L-phenylalanine was also higher in colorectal cancer compared to gastric cancer. In previously published data it was reported that some amino-acids including phenylalanine may be considered as a biomarkers in colorectal cancer patients (Hashim et al., 2019). Recently, Chen et al. (2022) presented that gut microbiome-associated serum metabolites can be used to detect colorectal cancer (Chen et al., 2022).

In the current study, it was observed higher grouping in case of gastric cancer in comparison to colorectal cancer, which confirms more homogeneous metabolic profile in gastric cancer patients. Moreover, on Scores Plot analysis there were created two subgroups in case of gastric cancer, such as Sample_1G and Sample_3G, Sample_4G and Sample_N2G. It may suggest similar characteristics in these two subgroups. Notably, the first subgroup created by Sample_1G and Sample_3G regards gastric cancer with similar TNM assessment (i.e. Sample_1G: T3N0M0, Sample_3G: T3N1M0). TNM tool is used to assess as follows: T – tumour, N – nodes (involvement of lymph nodes), and M – metastasis (Piñeros et al., 2019). The link between untargeted metabolomics in gastric cancer and both local and distal metastasis may exist, however it requires confirmation with larger sample size. In the current study, it was demonstrated that the metabolic profile of colorectal cancer patients is varied. It can be associated with different localisation of tumours – there are four cases analysed, i.e. sigmoid-rectal cancer, sigmoid colon cancer, rectal cancer, and ascending colon cancer.

## 5 Limitations and future directions

This study has been some limitations. First of all, the study was conducted with relatively small sample size. However, it is treated as preliminary results to present basic characteristics of untargeted metabolomics in gastric and colorectal cancer patients as well as to find the directions prior to the next study (KB/428-526/2023, Medical University of Gdansk, Gdansk, Poland) in which we analyse the impact of anti-cancer treatment regarding chemotherapy and radiotherapy on untargeted metabolomics aspects. This project is currently ongoing in cooperation with multi-disciplinary team of both oncologists and oncological surgeons. It is also recommended to investigate untargeted metabolomics among patients with similar both stage and grade of the cancers, nevertheless it might be challenging to collect stool samples with larger sample size. However, it would provide promising strategy to be included in clinical aspects. Metabolomics-based biomarkers might provide earlier detection of cancers allowing to complete resection of tumour. Moreover, metabolomics-related techniques can be attractive due to the fact that they are non-invasive and relatively low cost.

## 6 Conclusions

The aspect of untargeted metabolomics is a new area, which can be considered in oncology. Notably, the results presented in the current study were obtained prior to the introduction of anti-cancer management, such as surgical treatment. The overall insights into untargeted metabolomics showed that most often higher levels of analysed metabolites were detected in colorectal cancer patients compared to gastric cancer patients. It can be related to the different activity of gut microbiome in particular types of gastrointestinal cancer. Additionally, it was observed a higher grouping in case of gastric cancer comparing to colorectal cancer, which confirms more homogeneous metabolic profile in this cancer. The link between untargeted metabolomics in gastric cancer and both local and distal metastasis may exist, but it requires confirmation in further multi-centre studies regarding larger sample size.

## Data availability statement

The mass spectrometry proteomics data have been deposited to the ProteomeXchange Consortium via the PRIDE partner repository with the dataset identifier PXD051921.

## Ethics statement

The studies involving humans were approved by Independent Bioethics Committee for Scientific Research at the Medical University of Gdansk. The studies were conducted in accordance with the local legislation and institutional requirements. The participants provided their written informed consent to participate in this study.

## Author contributions

KK-S: Writing – review & editing, Writing – original draft, Resources, Project administration, Methodology, Investigation, Funding acquisition, Formal analysis, Conceptualization. DM: Writing – original draft. DS: Writing – review & editing, Software, Formal analysis. JM: Writing – review & editing. BS: Writing – review & editing, Data curation. PU: Writing – review & editing, Data curation. KP: Writing – review & editing. ES: Writing – review & editing, Supervision. KS-Z: Writing – review & editing, Supervision. LK: Writing – review & editing, Supervision, Conceptualization.
